# Genetically engineered orange petunias on the market

**DOI:** 10.1007/s00425-017-2722-8

**Published:** 2017-06-24

**Authors:** Hany Bashandy, Teemu H. Teeri

**Affiliations:** 1 Department of Agricultural Sciences, Viikki Plant Science Centre, University of Helsinki, P.O. Box 27, 00014 Helsinki, Finland; 2 Department of Genetics, Cairo University, 13 Gamaa St., Giza, 12619 Egypt

**Keywords:** Dihydroflavonol 4-reductase, Pelargonidin, Transgenic, *Petunia hybrida*

## Abstract

**Unauthorized genetically engineered orange petunias were found on the market.**

Genetic engineering of petunia was shown to lead to novel flower color some 20 years ago. Here we show that petunia lines with orange flowers, generated for scientific purposes, apparently found their way to petunia breeding programmes, intentionally or unintentionally. Today they are widely available, but have not been registered for commerce.

**Electronic supplementary material:**

The online version of this article (doi:10.1007/s00425-017-2722-8) contains supplementary material, which is available to authorized users.

## Introduction

The pathway to the colored anthocyanins in the ornamental plant petunia (*Petunia hybrida*) is a well-known example of substrate specificity of one enzyme limiting the spectrum of possible products of the pathway (Forkmann and Ruhnau 1987). Anthocyanins are water-soluble pigments giving flowers, fruits and sometimes vegetative parts of plants colors ranging from orange and red to blue and purple (Winkel-Shirley 2001). Anthocyanins are extensively glycosylated and acylated, the molecular decoration affecting their spectral properties. At the aglycone level the three most common variants of the molecule are the anthocyanidins pelargonidin, cyanidin and delphinidin, differing by the number of hydroxyl groups (one, two or three, respectively) in the B-ring of the molecule. Hydroxylation takes place at the level of dihydroflavonols in the pathway (possibly earlier in some cases, or later in others) by two enzymes, flavonoid 3′-hydroxylase (F3′H) and flavonoid 3′5′-hydroxylase (F3′5′H). The enzyme dihydroflavonol reductase (DFR) converts dihydroflavonols to corresponding leucoanthocyanidins, which then are oxidized to anthocyanidins by anthocyanidin synthase (syn. leucoanthocyanidin dioxygenase). In petunia, the DFR enzyme does not react with the simplest precursor (dihydrokaempferol); therefore, the natural range of petunia flower colors lacks orange hues typical to pelargonidin derivatives. Flowers of petunia cultivars that have mutations in the two hydroxylases are, therefore, white.

It was shown few decades ago that by introducing a gene encoding DFR from a species where the enzyme does not show substrate specificity into a petunia line that lacks F3′H and F3′5′H activity, one can open up the pathway to pelargonidin. Using the maize gene *A1* Meyer et al. (1987) generated brick red-colored flowers in petunia and using the gene from the ornamental plant *Gerbera hybrida*, our own laboratory generated petunia lines with bright orange flowers (Helariutta et al. 1993).

These petunia flowers were investigated concerning factors relating to stability of the transgene (and, therefore, the novel color; Meyer et al. 1992; Elomaa et al. 1995), but they were never commercialized. The list of registered genetically modified petunia plants is very short and includes a single line transgenic for a chalcone synthase encoding gene approved for cultivation in China (http://www.isaaa.org/gmapprovaldatabase/).

## Materials and methods

### Chemicals and plant material

Authentic standards for pelargonidin and cyanidin were purchased from TransMIT PlantMetaChem (Giessen, Germany). All other chemicals were from Sigma-Aldrich (Milano, Italy), water was purified by a Milli-Q water purification system. Petunia seeds were germinated and plants were grown in peat:vermiculite (1:1) under fluorescent illumination (16 h day length) at 23 °C. Origin of the different petunia cultivars is shown in Table 1, the nontransgenic line W80 (Huits et al. 1994) was a kind gift of Dr. Ronald Koes (University of Amsterdam, Swammerdam Institute for Life Sciences, Amsterdam, The Netherlands).

**Table 1:** “Orange-colored” petunias purchased for this study

Number	Cultivar name	Source	Main anthocyanidin	Comments
1	Cascadias Indian Summer	City of Helsinki	Cyanidin	Low amounts of anthocyanins
2	Bonnie Orange	City of Helsinki	Pelargonidin	
3	African Sunset	Amazon.com^a^	Pelargonidin	
4	African Sunset	Nicky’s Nursery^b^	Pelargonidin	
5	African Sunset	Etsy^c^		Did not germinate
6	African Sunset	Etsy^c^	Pelargonidin	
7	African Sunset	Swallowtail Garden^d^	Pelargonidin	
8	Aladdin Orange	Seedman^e^	Cyanidin	
9	Orange color Petunia	Aliexpress^f^		Flowers purple or white with purple stripes
10	Rare orange petunia	Aliexpress^f^		Did not germinate

Web sites for seed sources

^a^ https://www.amazon.com/African-Sunset-Beautiful-Orange-Petunia/dp/B00TG0E3PI

^b^ http://www.nickys-nursery.co.uk/garden-shop/seeds/hanging-baskets/petunia/petunia.-african-sunset-10-pellets

^c^ https://www.etsy.com/

^d^ http://www.swallowtailgardenseeds.com/annuals/petunia.html#gsc.tab=0

^e^ https://www.seedman.com/petunia.htm

^f^ http://www.aliexpress.com/price/orange-petunia-seeds_price.html

### RNA extraction, cDNA synthesis and RT-PCR reactions

Total RNA was isolated as described (Chang et al. 1993) from unopened flowers when anthocyanin biosynthesis was active and the petals were gaining color. A treatment with RNase-free DNase (Nucleo Spin RNA clean-up XS, Macherey–Nagel, Germany) was done to remove any residues of genomic DNA. First-strand cDNA was synthesized from 166 ng of total RNA using the Superscript III Reverse Transcriptase Kit (Invitrogen). Using the cDNA as template, the full-length maize *A1* transcript was amplified with primers GER945 and GER946, and a fragment of the *nptII* transcript with primers GER522 and GER523 using Phusion High-Fidelity DNA Polymerase (Thermo Scientific, Waltham, MA, USA). The thermal cycler was programmed in the following way: an initial cycle of denaturation at 98 °C for 30 s, followed by 25 (*A1*) or 30 (*nptII*) cycles of: 98 °C for 30 s, 65 °C (*A1*) or 56 °C (*nptII*) for 30 s, 72 °C for 1 min, followed by a final extension for 10 min at 72 °C.

### DNA extraction and genomic PCR

Genomic DNA was isolated from leaves using the miniprep II method (Dellaporta et al. 1983). 100 ng of petunia genomic DNA was used for amplification of sequences between the *bla* gene and the 35S promoter (primers GER1003 and GER992), between the 35S promoter and the maize *A1* gene (primers GER993 and GER984) and between the maize *A1* gene and the *nptII* gene (primers GER981 and GER976) with Dream Taq DNA polymerase (Thermo Scientific) with the following program: an initial cycle of denaturation at 95 °C for 3 min, followed by 30 cycles of: 94 °C for 1 min, 55 °C for 30 s, 72 °C for 2 min. Amplified fragments were purified using High Pure PCR Product Purification Kit (Roche Diagnostics, Indianapolis, IN, USA) and directly sequenced using the amplification primers and, when needed, internal primers designed from the sequences. The sequences were assembled together based on their overlapping segments and deposited in GenBank with the accession number KY964325.

### Anthocyanidin analysis

HPLC analysis was carried out as described (Bashandy et al. 2015) with minor modifications. Samples were collected from fully opened petunia flowers, ground in liquid nitrogen and extracted with a 2× volume of methanol with 1% HCl. After sonication for 30 min at room temperature (Finn sonic W181, Oy ULTRA sonic Finland Ltd, Lahti, Finland), the extracts were cleared from debris by centrifugation (3220*g*, 10 min). The supernatant was mixed 1:1 with 4 M HCl and hydrolyzed by incubating for 40 min at 95 °C. The hydrolyzed extracts were centrifuged at 17,000*g* for 10 min before injection into HPLC column. The standard compounds were applied at 10 µg/ml in methanol.

## Results and discussion

**Fig. 1:**
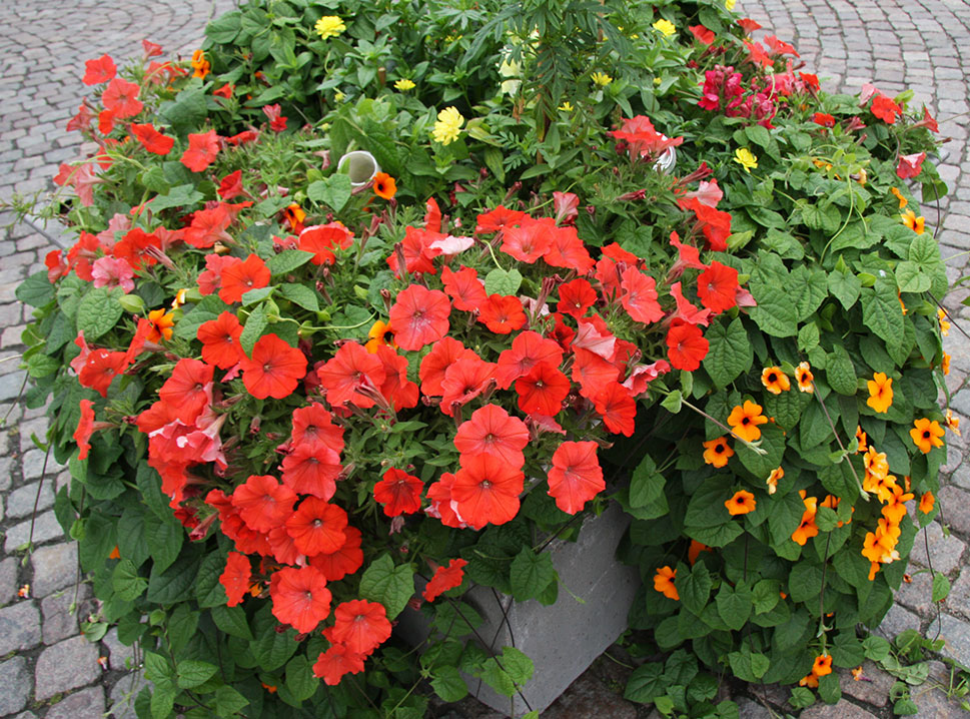
Orange petunias decorating the Helsinki railway station in 2016

Aware of the limitations in petunia color spectrum, it was a great surprise and a delight from the point of view of maybe gaining insight into the ways petunia germplasm changes under breeding, when we encountered bright orange-colored petunias in flower boxes decorating the Helsinki railway station during summers of 2015 and 2016 (Fig. 1). Indeed, orange petunias are widely on the market, as an internet search with these keywords shows. The cultivar at the Helsinki railways station was “Bonnie Orange”, for which we got a sample from the gardeners of the City of Helsinki in 2016. The cultivar “African Sunset” was available from several sources, along with a number of others that we were able to purchase (Table 1; Fig. S1).

Chemical analysis showed that while “Aladdin Orange” with orange red flowers was a cyanidin cultivar, “Bonnie Orange” and “African Sunset” unexpectedly contained pelargonidin as the main anthocyanidin. (Table 1; Fig. S2). Based on what we knew about petunia flower color and the history of orange petunias, we made a quick test using primers designed for the maize *A1* gene (using reverse transcription PCR) and saw that the orange flowers of “Bonnie Orange” and “African Sunset” indeed expressed a maize gene—and in addition the most common gene transfer selective marker gene *nptII* (Fig. S2). We further made a guess that these transgenic lines might contain the widely used CaMV 35S promoter sequence driving either of the two transgenes, and perhaps also the *bla* gene (for ampicillin resistance in bacterial hosts) common in many vectors. We designed primers that would amplify these sequences, as well as primers that would initiate amplification out of these sequences to nearby genetic elements (Table S1). Using an array of petunia chromosomal DNA samples (lines 1–4 and 6–9 in Table 1) and different combinations of the primers, we could amplify not only the *A1* and *nptII* genes, but also sequences between 35S and *A1*, *A1* and *nptII*, and *bla* and 35S. Amplification of these fragments took place repeatedly and exclusively from chromosomal DNA extracted from leaves of “Bonnie Orange” and “African Sunset” (Fig. S3). The PCR fragments were directly sequenced and due to the presence of overlapping parts they could be assembled into a single contig corresponding very precisely to the map presented in Meyer et al. (1987). We could not amplify the *bla* gene as a whole or sequences between the *bla* gene and the *nptII* gene, a possible explanation being that this region represents the integration site of the plasmid in a petunia chromosome. In fact, the line described in detail by Meyer et al. (1992) and used by Oud et al. (1995) in breeding experiments (see below) with a single copy insert and a truncated *bla* gene fits with these observations.

The retrieved sequences were identical in “Bonnie Orange” and “African Sunset”, indicating a common origin. Phenotypically these two cultivars are not exactly alike, for example, “African Sunset” has larger flowers than “Bonnie Orange”. The recipient line Meyer et al. (1987) used in their experiment was chosen based on its mutant genotype lacking both of the two hydroxylases (*ht ht hf hf*). It is not a line with good horticultural properties, but as with other interesting characters with simple inheritance, petunia breeders could introgress the gene for orange petal color to horticulturally superior genetic background by simple crossing. This was successfully done by Oud et al. (1995), showing that trait improvement by traditional breeding indeed works well also for genetically modified traits. The article ends by stating that “[the orange colour trait] has been successfully used in breeding programmes aimed at developing commercial F1 varieties with this trait”. This, relying on records, never happened. The regulations in Europe and elsewhere require an extensive analysis of risks a genetically modified organism might impose on human health and the environment upon deliberate release (i.e., commercialization). This expensive procedure prohibits the use of genetic modification in cases where the expected volume of production would be too small to cover the extra expenses—obviously the case for a petunia cultivar with a novel color.

The orange petunia lines generated for scientific purposes apparently found their way to petunia breeding programmes, intentionally or unintentionally. This particular escaped genetically engineered (GM) line causes no harm, but demonstrates that containment procedures can never be made failproof. A more important observation is that regulation of GM crops based on the breeding method instead of the cultivar’s properties in practice completely prohibits commercialisation of lines with traits that have beneficial but only incremental value—the typical pattern of gain in plant breeding. Although orange-colored petunias are mere examples of breeding for beauty, for developing better crops the lost opportunities of GM breeding have much wider consequences.

### *Author contribution statement*

TT conceived and designed the research. HB and TT conducted the experiments and analyzed the data. TT wrote the manuscript, which HB read and approved.

## Electronic supplementary material

Below is the link to the electronic supplementary material.

Supplementary material 1 (PDF 455 kb)
