# Supplementary material for: Genetically engineered orange petunias on the market
Source: Planta. 2017 Jun 24;246(2):277–80. doi: 10.1007/s00425-017-2722-8 (PMC5522655; doi:10.1007/s00425-017-2722-8)
Supplement: Supplementary file 1 — Supplementary material 1 (PDF 455 kb) [file 425_2017_2722_MOESM1_ESM.pdf]

Genetically engineered orange petunias on the market

Hany Bashandy and Teemu H. Teeri

Supplementary material

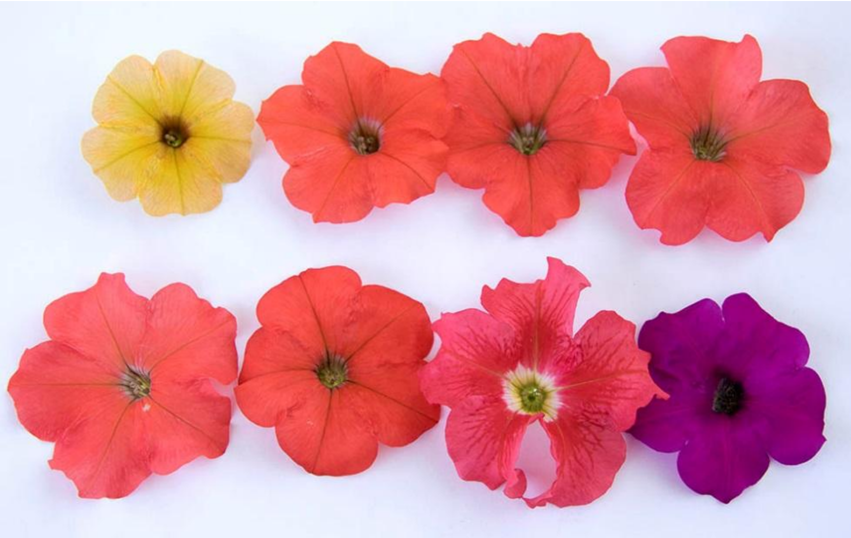

Fig. S1. Petunia cultivars “Indian Summer”, “Bonnie Orange”, “African Sunset” (4 sources), “Aladdin Orange” and “Orange color petunia”.

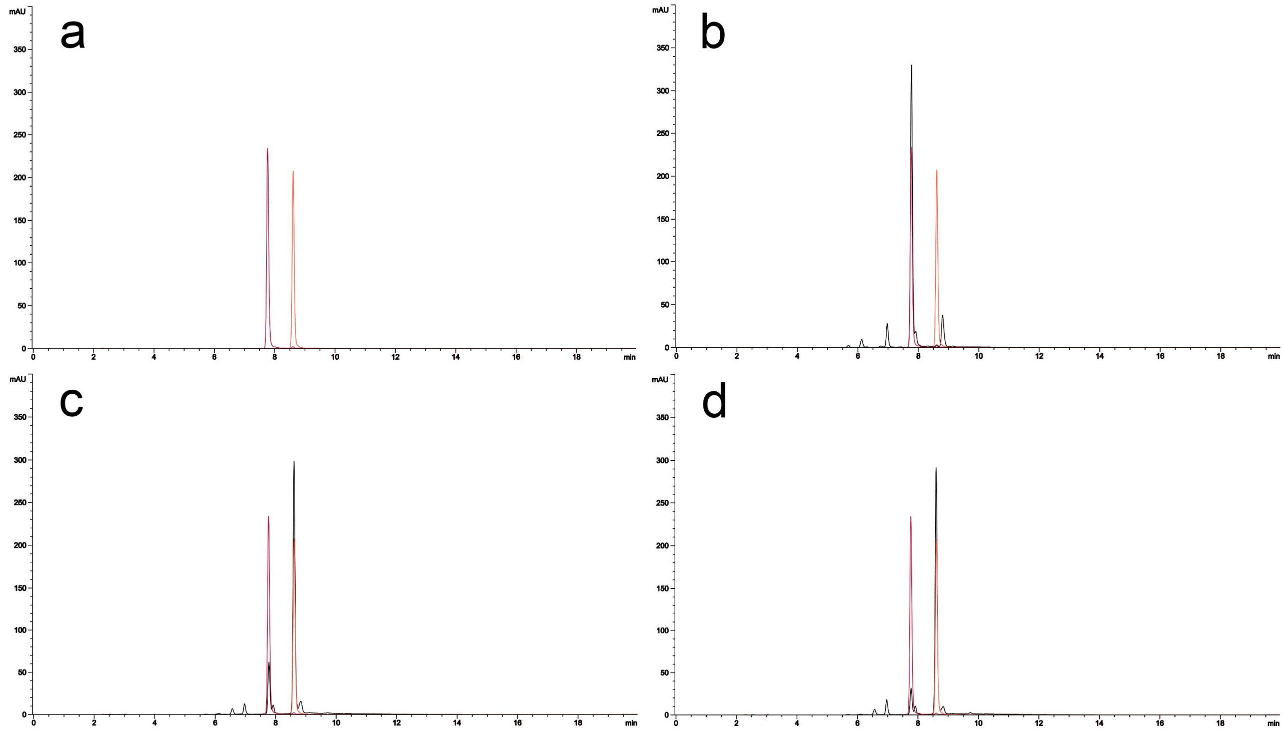

Fig. S2. HPLC chromatograms of anthocyanidins extracted from orange petunia flowers.
(**a**). Authentic standards for cyanidin (left, 7.80 min) and pelargonidin (right, 8.64 min).
(**b**). Cultivar ”Aladdin Orange”, containing cyanidin derived anthocyanidins.
(**b**). Cultivar ”Bonnie Orange”, containing pelargonidin derived anthocyanidins.
(**d**). Cultivar ”African Sunset”, containing pelargonidin derived anthocyanidins.
Panels B to D are overlayed with the chromatograms of the authentic standards (in color).

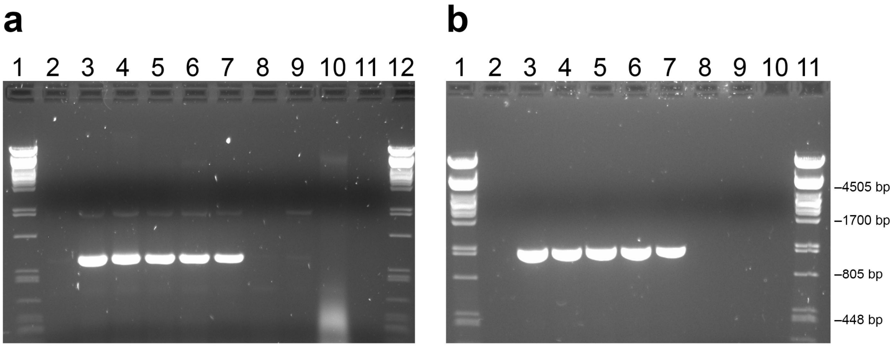

Fig. S3. RT-PCR from petunia mRNA. Outermost lanes: PstI digested Lambda DNA.
(**a**) Primers amplifying *nptII* sequences. Lanes 2-11: “Cascadias Indian Summer”, “Bonnie Orange”, “African Sunset” (four sources), “Alladin Orange”, “W80”, “Orange color Petunia”, water.
(**b**) Primers amplifying the maize *A1* DFR sequences. Lanes 2-10: “Cascadias Indian Summer”, “Bonnie Orange”, “African Sunset” (four sources), “Alladin Orange”, “W80”, water.

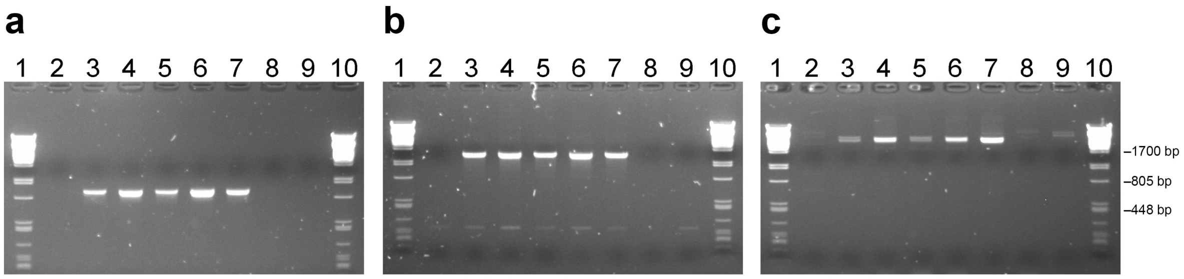

Fig. S4. PCR from petunia genomic DNA. Outermost lanes: PstI digested Lambda DNA. Lanes 2-9: “Cascadias Indian Summer”, “Bonnie Orange”, “African Sunset” (four sources), “Alladin Orange”, “Orange color Petunia”.
(**a**) Primers amplifying sequences from *bla* to the 35S promoter.
(**b**) Primers amplifying sequences from the 35S promoter to the maize *A1* DFR.
(**c**) Primers amplifying sequences from the maize *A1* DFR to *nptII*.

**Table S1. Primers used in this study**		
**Name**	**Sequence**	**Description**
GER945	AAAAAGCAGGCTCCATGGAGGGAGGTGCCG	Forward primer at start of the maize *A1* open reading frame
GER946	AGAAAGCTGGGTTAAGCGCCAATCGTCG	Reverse primer at end of the maize *A1* open reading frame
GER522	TCAAGACCGACCTGTCCGGT	Forward primer at 167 bp of *nptII* open reading frame
GER523	GAGGAAGCGGTCAGCCCATT	Reverse primer at 744 bp of *nptII* gene open reading frame
GER1003	ATAATACCGCGCCACATAGC	Reverse primer at 178 bp of the *bla* open reading frame
GER992	GTGCGTCATCCCTTACGTCA	Reverse primer 63 bp upstream of transcription start in 35S promoter
GER993	AGAACTCGCCGTGAAGACTG	Forward primer 493 bp upstream of transcription start in 35S promoter
GER984	TCGGGGTACCTATCCCTGAG	Reverse primer at 833 bp of the maize *A1* cDNA open reading frame
GER981	CTTCGTCGGCTCCTGGCTC	Forward primer at 60 bp of the maize *A1* cDNA open reading
GER976	GAAGAACTCGTCAAGAAGGCG	Reverse primer at 801 bp of the *nptII* gene open reading frame
